# Fatal Zargar grade 3b corrosive injury after hydrochloric acid ingestion: A case report

**DOI:** 10.1097/MD.0000000000040017

**Published:** 2024-10-04

**Authors:** Ching-Hsiang Yu, Yu-Jang Su, Yen-Chun Lai

**Affiliations:** a Department of Emergency Medicine, MacKay Memorial Hospital, Taipei, Taiwan; b Toxicology Division, Department of Emergency Medicine, MacKay Memorial Hospital, Taipei, Taiwan; c Department of Medicine, MacKay Medical College, New Taipei City, Taiwan; d Department of Nursing, MacKay Junior College of Medicine, Nursing, and Management, Taipei City, Taiwan; e Department of Nursing, Yuanpei University of Medical Technology, Hsinchu, Taiwan; f Department of Anesthesiology, Taipei Medical University Hospital, Taipei City, Taiwan.

**Keywords:** acidosis, case report, hydrochloric acid, perforation, shock

## Abstract

**Rationale:**

Caustic substance ingestion is an emergency and life-threatening condition as it leads to tissue damage, acidosis, and multiorgan failure. This study presents a case report of hydrochloric acid ingestion and notably dark-red urine output due to acute tubular necrosis.

**Patient concerns:**

A 59-year-old male presented with attempted suicide by ingesting 500 mL of hydrochloric acid (37%), and complained of severe abdominal pain and shortness of breath. Upon arrival, his vital signs showed a temperature of 34.3°C, blood pressure of 104/77 mm Hg, a pulse rate of 135 beats per minute, and the Glasgow Coma Scale E4V2M6. Following Foley catheter insertion, dark, bloody urine resulting from acute tubular necrosis was observed. His creatinine level was 1.1 mg/dL, and urinalysis showed 38 red blood cells per high-power field. Arterial blood gas analysis revealed metabolic acidosis.

**Diagnoses:**

The patient’s condition rapidly deteriorated in the emergency room, revealing diffuse circumferential ulceration with necrosis in the esophagus (Zargar score grade 3b). An exploratory laparotomy was performed for acidosis with intractable shock, revealing up to 1500 mL of bloody ascites, and ischemic changes with loss of peristalsis throughout the small bowel to the cecum.

**Interventions:**

Esophagostomy with T-tube insertion was performed. Notably, stomach necrosis with perforation was identified, prompting a surgical consultation for primary perforation closure.

**Outcomes:**

During the operation, the patient experienced hemodynamic instability. The family confirmed the “Do Not Resuscitate” status, and he died in a critical state.

**Lessons:**

For corrosive injuries, early endoscopy was crucial in assessing the extent of the damage and guiding treatment in this patient. It is essential to perform an early endoscopic examination in cases of acute nephrotoxic tubular necrosis following hydrochloric acid ingestion. Surgical intervention is warranted if necrosis is detected in the corrosive tissue.

## 1. Introduction

Caustic substance ingestion is a life-threatening emergency in daily practice that causes tissue damage, acidosis, and multiorgan failure.^[1]^ Herein, we report a case of hydrochloric acid ingestion and dark-red urine output due to acute tubular necrosis. We also discuss urine discoloration in the emergency department. Urine discoloration is clinically impressive and is often an indicator of diagnosis in emergency medicine. Although the toxic exposure history in this case is quite clear, abnormal urine color in the emergency department is often a clinical diagnostic clue as well. Therefore, in the discussion, we also included various phenomena and potential diagnoses associated with abnormal urine color in emergency patients, broadening our perspective and observations.

## 2. Case report

**Figure 1.**
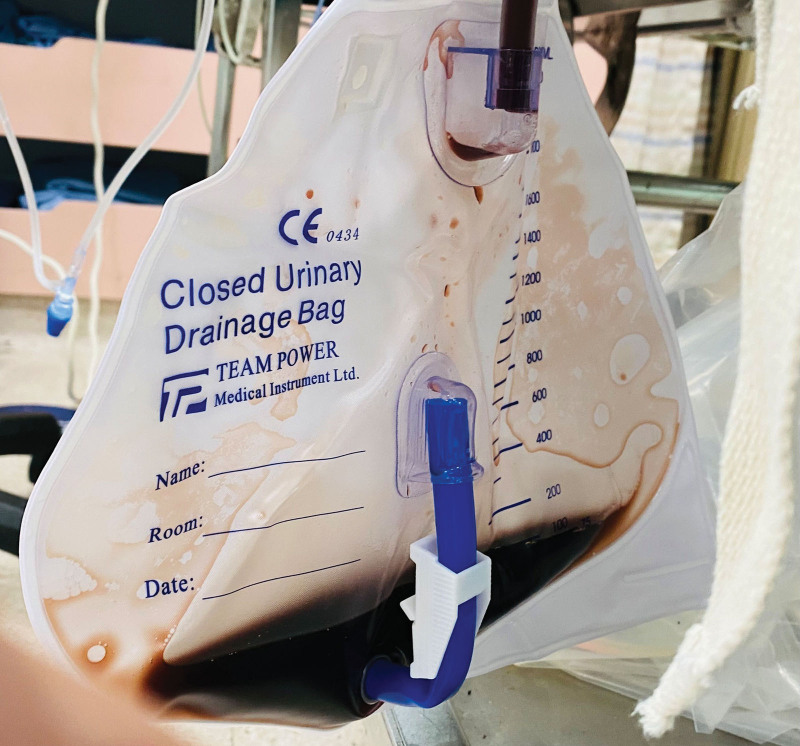
Dark, bloody urine resulting from acute tubular necrosis was observed.

A 59-year-old male with an unremarkable medical history attempted suicide by ingesting 500 mL of hydrochloric acid (37%) and presented to our emergency department. He complained of severe abdominal tenderness and shortness of breath, concurrent intense pain in the mouth and throat, hoarseness, and difficulty in swallowing. Upon arrival, his vital signs showed a temperature of 34.3°C, blood pressure of 104/77 mm Hg, a pulse rate of 135 beats per minute, and a respiratory rate of 29 breaths per minute. His Glasgow Coma Scale score was E4M6V2. Physical examination revealed an eroded oral cavity and diffuse abdominal tenderness with positive peritoneal signs. Additionally, dark, bloody urine was observed (Fig. 1) following Foley catheter insertion; his creatinine level was 1.1 mg/dL, serum potassium was 5.1 meq/L, and urinalysis showed 38 red and 2 white blood cells per high-power field. His initial arterial blood gas analysis on 3L nasal cannula showed metabolic acidosis (pH 7.130, pCO_2_ 23 mm Hg, pO_2_ 140 mm Hg, HCO_3_ 8 mmol/L, base excess −19.8 mmol/L, SaO_2_ 98.3%); 440 mL of 7% NaHCO_3_ was administered. Computed tomography demonstrated esophageal wall edema indicative of corrosive esophageal injury, ground-glass infiltrates in the right lower lobe, and focal consolidations in the bilateral lower lobes. Left-sided pleural effusion and ascites were observed.

Nasogastric tube decompression was performed immediately. Parenteral antibiotics were administered, including teicoplanin 400 mg, cefoperazone 1 g, sulbactam 1 g, and metronidazole 500 mg. The patient’s condition rapidly deteriorated in the emergency room, with systolic blood pressure as low as 42 mm Hg. Aggressive treatment with 1500 mL of lactated Ringer challenge, 200 mL of 25% albumin, 8 mg of norepinephrine in 250 mL of 5% dextrose given at 10 mL/h, and vasopressin 40 IU in 100 mL of 5% dextrose at 2 g/h were administered.

## 3. Diagnosis

**Figure 2.**
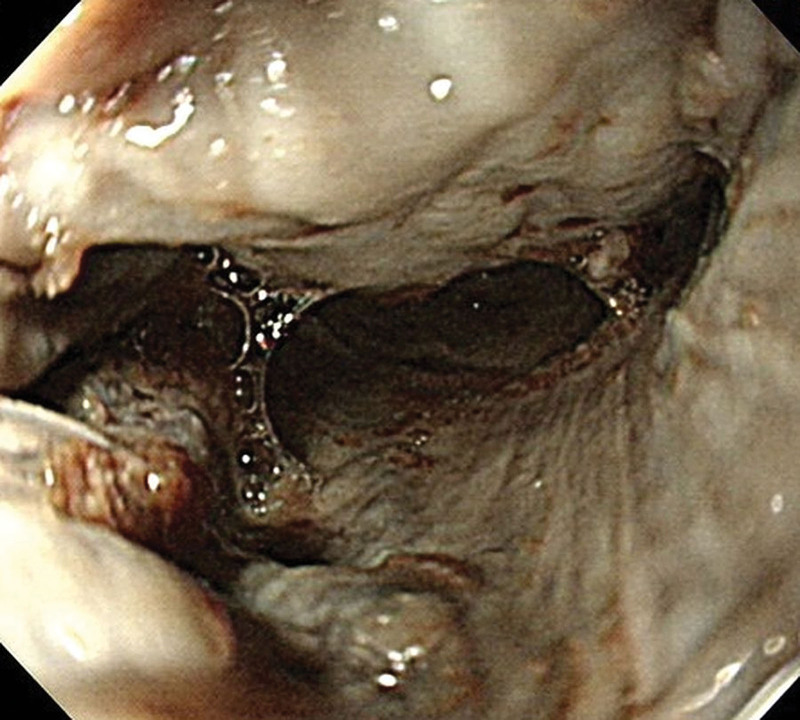
Diffuse circumferential ulceration with necrosis in the esophagus (Zargar score grade 3b).

**Figure 3.**
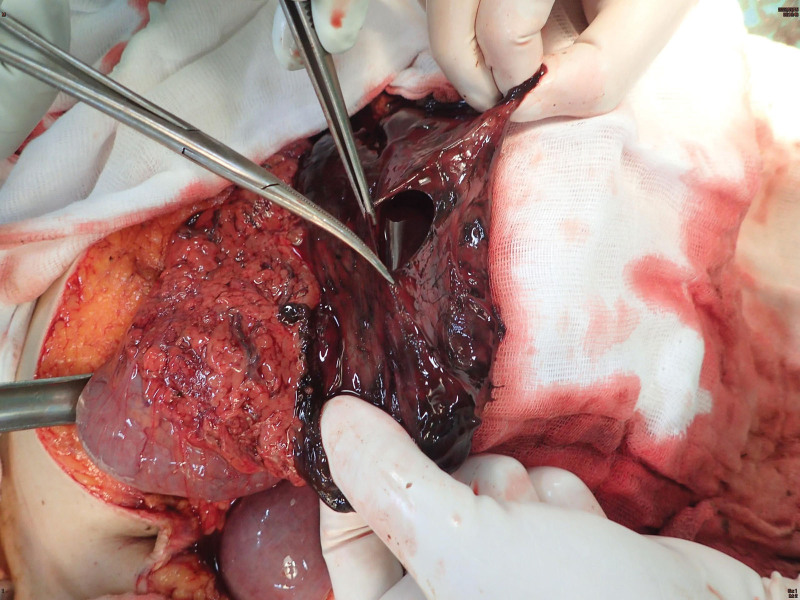
Body of stomach necrosis with perforation was found intraoperatively.

A nephrology consultation was sought because of refractory metabolic acidosis. A consulting gastroenterologist performed an emergency pan-endoscopy, revealing diffuse circumferential ulceration with necrosis in the esophagus (Zargar score grade 3b) (Fig. 2). Severe stricture of the upper esophagus, 20 cm from the oral incisor, precluded further advancement of the endoscope. Owing to this endoscopy finding, an exploratory laparotomy was performed for acidosis with intractable shock, unveiling up to 1500 mL of bloody ascites, and ischemic changes with loss of peristalsis throughout the small bowel to the cecum. Esophagostomy with T-tube insertion was performed. Notably, stomach necrosis with perforation (Fig. 3) was identified, prompting surgical consultation for primary closure of the perforation.

A thoracic surgeon performed a laparotomy via an upper midline incision and discovered necrosis with perforation of the stomach. A general surgeon, who repaired the perforation with primary closure, was then consulted. The thoracic surgeon subsequently performed an esophagostomy with T-tube insertion in the lower esophagus via an abdominal approach. The patient was hemodynamically unstable intraoperatively. After discussing with his family the grave prognosis, “Do Not Resuscitate (DNR)” was confirmed; the patient expired on the following day in a critical state.

## 4. Discussion

This is the first study to describe suicidal hydrochloric acid ingestion, urine discoloration, outcomes, and the clinical course. Caustic injuries are more common in females (54.1%) in comparison to males (45.9%), according to a 2021 report from Northern Taiwan.^[2]^ Strong caustic substances account for 65.6% of ingested items.^[2]^ A Turkish study (n = 2578) reported that the majority of corrosive substance intake cases were accidental; however, 16.2% of cases were suicidal intentions. Alkaline corrosive substances were more common than acidic substances (60.8% vs 36.5%, respectively). Half of the corrosive substance intake was from cleaning solutions, such as sodium hypochlorite. After primary treatment in the emergency department, it took approximately 4 hours to be hospitalized. Zargar score grade 1 is the most common endoscopic finding, accounting for 35.6% of cases.^[3]^

In cases of clinical practice in the emergency department, urine discoloration was usually noted to raise concern in situations of infection or poisoning (Table 1^[4–7]^). These observations of abnormal urine color also provide insights into the possible clinical events occurring in the patient, aiding in diagnosis.

**Table 1:** Urine discoloration in the emergency department.

Urine color	Disease	Mechanism	Reference
Dark-red	Suicide by hydrochloric acid ingestion	Acute tubular necrosis	This case
Black	Cresol intoxication	Presence of phenolic metabolites	[4]
Black, red, and orange urine sequentially	Quinalphos Poisoning	The decreasing severity of hemolysis has probably contributed to the change in urine color from black to brown and subsequently to orange	[5]
Purple	Urinary tract infection	Tryptophan is metabolized by intestinal bacteria, after which the by-product indoxyl sulfate is expelled into the urine and digested into indoxyl by sulfatases/phosphatases produced by bacteria. This indoxyl may convert into indigo and indirubin in the urine drainage bag and create purple discoloration	[6,7]

The presence of dark urine in this case signifies gross hematuria, suggestive of acute kidney injury or acute tubular necrosis. This condition may arise due to ischemia secondary to shock and reduced circulating blood volume. Alternatively, tubular obstruction by-products absorbed from the damaged esophageal and gastric mucosa, particularly hemoglobin, may contribute to renal dysfunction.^[8]^ Hence, acute kidney failure represents an additional risk factor in patients with endoscopic grade 3 mucosal injury, necessitating urgent intervention despite the absence of perforation.^[9]^

In this patient, early endoscopy for corrosive injuries helped assess the extent of the injury and its disposal. A Taiwanese study concluded that computed tomography is necessary to evaluate the extent or detect perforated injuries in seriously injured patients. However, endoscopy cannot replace mild-to-moderate injuries.^[10]^ A 20-year prospective report from northern Taiwan (n = 468) described caustic substance ingestion with risks of mortality and perforation in dose- and pH-dependent alkalis. The severe endoscopic findings (Zargar grade ≥2b) were pH-dependent for both substance types, and additionally dose-dependent for acids. The overall mortality rate was 3.6%, and all fatal cases were Zargar 2b and 3 (17, 3.6%).^[1]^ In another 14-year Spanish report on 21 cases of hydrochloric acid ingestion, 17 (81%) patients did not undergo endoscopic examination, 8 (38.1%) patients underwent surgical interventions, and 14 (66.7%) patients died.^[11]^

## 5. Conclusions

Hydrochloric acid ingestion results in dark-red urine and life-threatening conditions. We presented an impressive urine color and clinical course; although aggressive treatment was applied, the patient died after surgical intervention. Early endoscopic examination is mandatory for caustic injury and surgical intervention is required if the corrosive tissue shows necrosis.

## Acknowledgments

The authors thank Doctor Hsiu-Wu Yang’s for gathering data (laboratory data).

## Author contributions

**Data curation:** Ching-Hsiang Yu, Yu-Jang Su.

**Formal analysis:** Ching-Hsiang Yu.

**Investigation:** Ching-Hsiang Yu, Yu-Jang Su.

**Resources:** Ching-Hsiang Yu, Yu-Jang Su.

**Writing—original draft:** Ching-Hsiang Yu, Yu-Jang Su.

**Conceptualization:** Yu-Jang Su, Yen-Chun Lai.

**Methodology:** Yu-Jang Su, Yen-Chun Lai.

**Project administration:** Yu-Jang Su.

**Supervision:** Yu-Jang Su, Yen-Chun Lai.

**Writing—review & editing:** Yu-Jang Su, Yen-Chun Lai.

**Visualization:** Yen-Chun Lai.
